# Risk assessment of acute heart failure after endovascular therapy in acute ischemic stroke: a nomogram-based study

**DOI:** 10.3389/fcvm.2026.1836280

**Published:** 2026-07-20

**Authors:** Xiuling Yang, Wenfei Liang, Shaoan Ge, Bolin Song, Jingling Zhu, Yu Ding, Yilin He, Zhan Zhao, Bin Liao, Weimin Ning, Qiuxing He, Jingyi Chen

**Affiliations:** 1 Department of Neurology, Dongguan Hospital of Guangzhou University of Chinese Medicine, Dongguan, China; 2 Dongguan Key Laboratory of Intractable Brain Diseases in Dongguan, Dongguan Hospital of Guangzhou University of Chinese Medicine, Dongguan, China; 3 State Key Laboratory of Dampness Syndrome of Chinese Medicine, Dongguan Hospital of Guangzhou University of Chinese Medicine, Dongguan, China

**Keywords:** acute heart failure, acute ischemic stroke with large vessel occlusion, endovascular therapy, nomogram, risk prediction

## Abstract

**Background:**

Acute heart failure (AHF) is a life-threatening complication following endovascular therapy (EVT) for acute ischemic stroke with large vessel occlusion (AIS-LVO). This study aims to identify independent predictors of AHF after EVT and to develop and validate a nomogram for individualized risk prediction.

**Methods:**

We retrospectively enrolled data from patients with acute ischemic stroke with large vessel occlusion who underwent endovascular treatment between September 2021 and June 2025 were analysed in October 2025. After screening based on inclusion and exclusion criteria, 457 patients were randomly allocated into training (*n* = 320) and validation (*n* = 137) cohorts in a 7:3 ratio. Least absolute shrinkage and selection operator (LASSO) regression was used for feature selection, followed by multivariate logistic regression to identify independent predictors of AHF. A nomogram was constructed accordingly. The model's performance was assessed using the area under the receiver operating characteristic curve (AUC), calibration plots, decision curve analysis (DCA), and clinical impact curves (CIC).

**Results:**

Multivariate analysis identified five independent predictors of AHF after EVT: atrial fibrillation, valvular heart disease, left ventricular ejection fraction, serum creatinine, and troponin T. The nomogram demonstrated excellent discriminative ability, with an AUC of 0.909 (95% CI: 0.837–0.980) in the validation cohort. Calibration curves indicated good agreement between predicted and observed risks. DCA and CIC confirmed the model's favorable clinical net benefit.

**Conclusion:**

We developed and validated a five-factor nomogram that effectively predicts the individualized risk of AHF following EVT in patients with AIS-LVO.

## 1 Introduction

Stroke is one of the main causes of disability and the second predominant cause of death worldwide (1), imposing a significant socio-economic burden (2). Acute ischemic stroke with large vessel occlusion (AIS-LVO) is one of the most disabling and fatal subtypes of ischemic stroke (3). Endovascular treatment (EVT), as the standard therapeutic approach for LVO patients, can significantly improve vascular recanalization rates and neurological outcomes (4). However, stroke patients often develop a severe complication—acute heart failure (AHF)—due to exacerbation of underlying cardiovascular diseases, hemodynamic fluctuations, and postoperative systemic inflammatory responses (5). This condition not only prolongs hospital stays but also greatly raises the risk of mortality within three months after the procedure (6).

AHF refers to a group of clinical syndromes characterized by a sudden decline in the heart's ability to contract or relax, leading to a reduction in cardiac output insufficient to fulfill systemic metabolic requirements. This results in pulmonary or systemic congestion, as well as inadequate perfusion of tissues and organs (7). Approximately 20% of AIS patients in a randomized trial have significant cardiac problems (8), which include heart failure. These severe cardiac problems are even more frequent in patients who have had a major stroke (5).

However, the current diagnostic and therapeutic approaches to acute heart failure in post-stroke patients face several challenges. Stroke patients frequently have comorbid conditions such as atrial fibrillation and atherosclerosis (9), making it difficult to accurately identify high-risk heart failure patients using traditional single-indicator methods. Early identification of acute heart failure after stroke is crucial; failure to promptly diagnose and manage this condition may delay subsequent treatment, leading to a series of adverse consequences. Furthermore, patients who experience new cardiovascular problems following a stroke have a higher than 50% chance of having another stroke within five years, and their five-year mortality rate is significantly higher (10). Therefore, early detection of AHF-risk individuals in AIS-LVO patients following EVT, and the timely implementation of targeted interventions, is of critical clinical significance for improving overall patient outcomes.

Currently, research on predicting AHF after EVT for LVO remains in its early stages. Most existing studies focus on the epidemiological characteristics of stroke patients with heart failure, but lack comprehensive, multi-factorial, quantitative prediction tools. Nomograms, as visual clinical prediction models, can integrate multiple independent risk factors into an intuitive risk scoring system. They offer advantages such as ease of use and clear predictive efficacy. However, there is currently no nomogram model available for predicting AHF risk in patients with AIS-LVO after EVT, making it challenging for clinicians to appropriately analyze the risk of AHF among these patients.

## 2 Methods

### 2.1 Study design and participants

This study aims to retrospectively analyze the clinical data of patients undergoing EVT for LVO, identify independent risk factors for post-procedural AHF, and construct a multi-factorial nomogram prediction model. Through internal validation, we will assess the model's discriminatory ability and calibration, intending to give medical professionals a straightforward and trustworthy risk assessment tool to ensure early identification and tailored care of patients with a severe risk of AHF following EVT. Ultimately, this model aims to decrease the frequency of complications and enhance the prognosis of patients.

This research was approved by the Institutional Review Board of Dongguan Hospital of Chinese Medicine [No. PJ (2025) 135] and was conducted in compliance with the Declaration of Helsinki. Given that the research was retrospective and based on an existing database, and the study process did not involve direct intervention with participants. It met the ethical review requirements and was granted an exemption from obtaining informed consent by Institutional Review Board of Dongguan Hospital of Chinese Medicine.

At the Dongguan Hospital of Traditional Chinese Medicine, a single-center, retrospective, observational cohort study was conducted. A total of 457 patients diagnosed with AIS-LVO who underwent EVT between September 2021 and June 2025 were enrolled, and the data were analysed in October 2025. The primary outcome of the study was the incidence of in-hospital AHF, and the AHF events were adjudicated according to established clinical diagnostic criteria. The primary outcome was independently reviewed by two experienced senior physicians who evaluated all available medical records and diagnostic examination results.

Inclusion Criteria: (1) Diagnosis of AIS-LVO, confirmed according to the *2019 AHA/ASA Guidelines* (11) for the Early Management of Acute Ischemic Stroke, and received endovascular treatment. (2) AHF diagnosed according to the *2023 European Society of Cardiology (ESC) Guidelines for the Diagnosis and Treatment of Acute Heart Failure* (Updated Version) (12). ①Typical Symptoms of AHF: such as acute dyspnea, cyanosis, lower limb edema, coughing up pink and frothy sputum; ②Typical signs AHF: pulmonary rales, gallop rhythm, peripheral edema, and hepatomegaly; ③Objective evidence of cardiac structural or functional abnormalities: electrocardiogram, pulse oximetry, echocardiography, initial laboratory investigations, and other specific evaluations. ④Abnormal natriuretic peptide levels: NT-proBNP≥300 pg/mL [>450 pg/mL if under 55 years, >900 pg/mL if between 55 and 75 years, and >1800 pg/mL if above 75 years (13)]. For patients with concomitant atrial fibrillation or renal failure, the BNP diagnostic threshold should be increased by approximately 20%–30%.

Exclusion Criteria: (1) Patients who did not undergo EVT; (2) Patients diagnosed with transient ischemic attack (TIA); (3) Patients who had AHF prior to admission or before undergoing surgery; (4) Patients with incomplete clinical data; (5) Pneumonia predominantly caused by pulmonary infection, neurogenic pulmonary edema, respiratory dysfunction and other non-cardiogenic acute respiratory failure events; (6) isolated volume overload without clinical or imaging evidence of heart failure; (7) other severe complications that might confound the clinical diagnosis. Ultimately, 457 patients who underwent EVT for acute ischemic stroke were included in the study. During hospitalization, patients were randomly assigned in a 7:3 ratio to a training cohort (*n* = 320) and a validation cohort (*n* = 137).

### 2.2 Predictor variable collection

Based on literature review and clinical experience, 24 readily obtainable clinical variables were selected. The following information was systematically extracted from patients’ electronic medical records: (1) Demographic characteristics: age, sex, medical history (hypertension, diabetes mellitus, chronic obstructive pulmonary disease, atrial fibrillation), and vital signs [admission systolic and diastolic blood pressure (14)]. (2) Laboratory indices: hemoglobin, C-reactive protein (CRP), high-density lipoprotein, low-density lipoprotein, D-dimer, potassium, as well as cardiac troponin T (cTnT) and creatinine (Cr), all defined as the first measurements obtained within 24 h after admission. (3) Imaging indices: left ventricular ejection fraction (LVEF) and systolic and diastolic cardiac function, all assessed by transthoracic echocardiography (TTE) within 24 h of admission, and ST-segment changes assessed by 12-lead electrocardiogram (ECG) monitoring performed for all patients. (4) Neurological deficit assessment: The National Institutes of Health Stroke Scale (NIHSS) was employed (15), it consists of 15 items with a total score range of 0–42; scores <5 indicate mild deficit, scores 5–15 indicate moderate deficit, and scores ≥16 indicate severe deficit. (5) Glasgow Coma Scale (GCS) (16): GCS scores of 13–15 indicate mild brain injury, 9–12 indicate moderate brain injury, and 0–8 indicate severe brain injury. (6) Modified Rankin Scale (mRS) (17): The mRS was used as the standardized measure of global disability outcome,it comprises seven levels (0–6); scores of 0–3 indicate a favorable functional status (independent in basic activities of daily living), whereas scores of 4–6 indicate functional impairment (not fully independent in daily living).

### 2.3 Analysis of statistics

Statistical analysis was primarily accomplished using R (4.5.1) and SPSS (31.0). Missing data were handled using multiple imputation by chained equations (MICE). Covariates with a missing rate of less than 20% were eligible for imputation. Descriptive statistics were performed for all 457 participants. Frequencies (n) and percentages (%) were used to summarize categorical data, and the *χ*^2^ test was employed to compare groups. The normality of continuous variables was evaluated; normally distributed data were reported as mean ± SD (x¯ ± s), and intergroup comparisons were performed using the independent samples t-test. Non-normally distributed data as median (Q1, Q3) [M (Q1, Q3)], the Mann–Whitney U test was employed for group comparisons. A *P*-value <0.05 was deemed statistically significant, and all statistical tests were two-tailed.

After data cleaning and missing-data imputation were completed. To ensure reproducibility, the dataset was randomly split into training and validation cohorts in a 7:3 ratio using the sample function in R with a predefined random seed (125). The suitable penalty parameter (*λ*) was found using tenfold cross-validation, and 500 bootstrap resamples were applied for bias-corrected internal validation to assess model stability and generalizability. The variance inflation factor (VIF) was first used to assess multicollinearity in all possible predictors; VIF＜10 indicated the absence of severe multicollinearity. Feature selection was performed using LASSO regression, where *λ* was tuned based on the cross-validation error minimum within one standard error (*λ*.1se ≈ 0.05) was chosen as the optimal penalty parameter. A multivariable logistic regression model was then created using the variables chosen via LASSO, and multicollinearity was reassessed to ensure stability of parameter estimates. Model discrimination was evaluated using the receiver operating characteristic (ROC) curve and the area under the curve (AUC), while goodness of fit and calibration were assessed using the Hosmer–Lemeshow test, calibration curves, and the Brier score. Decision curve analysis (DCA) and clinical impact curves (CIC) were further applied to quantify the net clinical benefit across different threshold probabilities, thereby determining the clinical utility of the model.

## 3 Results

### 3.1 Flowchart

**Figure 1:**
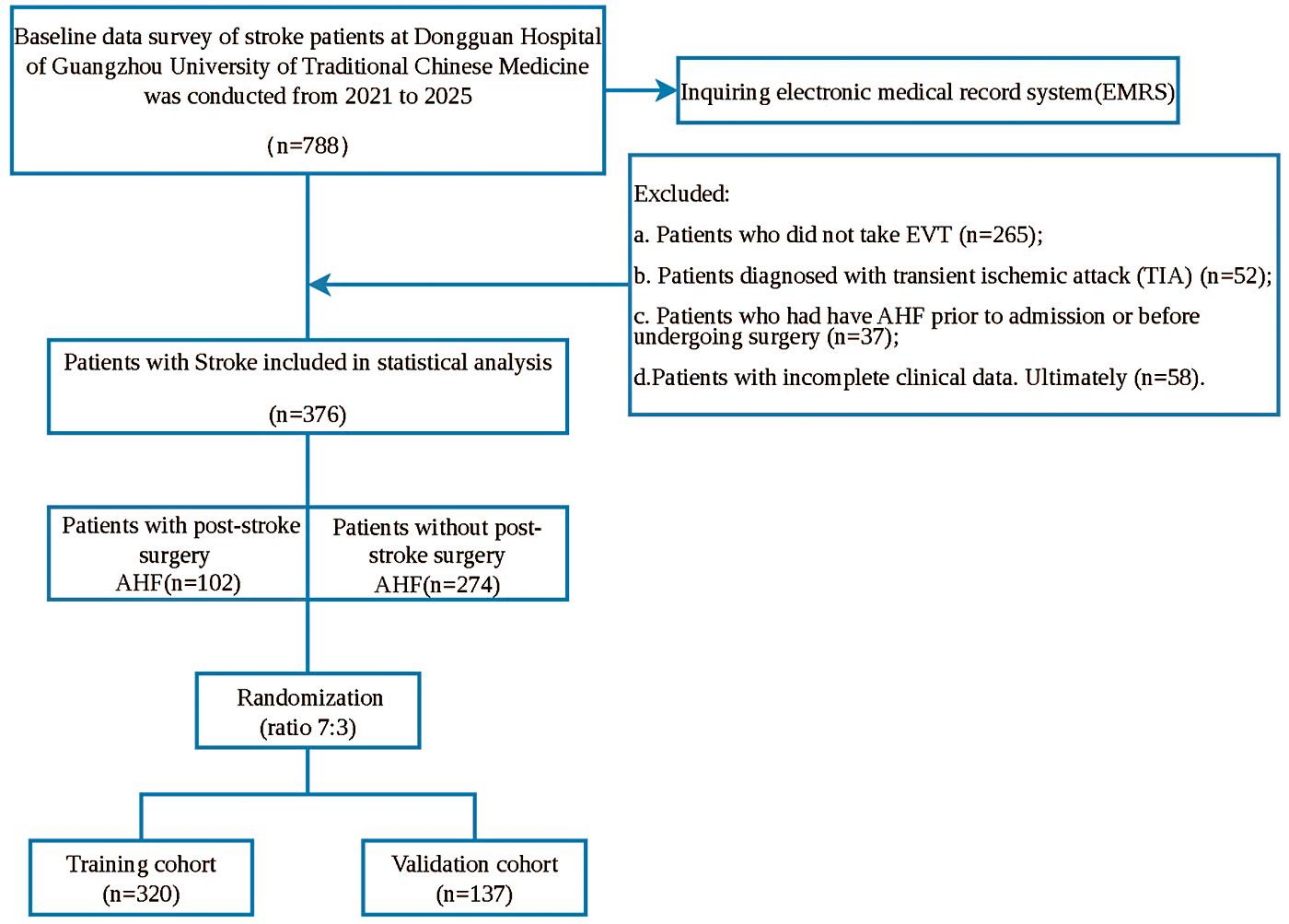
The flowchart of the AHF after endovascular therapy in patients with acute large vessel occlusion stroke.

All variables had a missing rate of less than 20% and were therefore included in the analysis (Supplementary Figure S1). The study Procedure diagram is shown in Figure 1. Among the 788 patients initially screened for eligibility, 331 were excluded. Ultimately, AHF was found in 102 (22.32%) of the 457 individuals in the analysis following EVT for AIS-LVO, and 355 (77.68%) were classified as non-AHF. Based on a 7:3 random sampling ratio, the training cohort consisted of 320 individuals, while the validation cohort comprised 137.

### 3.2 Patient characteristics

This study comprised 457 patients in total, split into two groups: 102 patients (22.32%) in the post-stroke acute heart failure (AHF) group and 355 patients (77.68%) in the non-AHF group. Table 1 highlights the variations between the two groups regarding demographic information, clinical traits, laboratory markers, and pertinent scoring scales.

**Table 1:** Comparative analysis of baseline characteristics between training and validation cohorts.

Variables		Training Cohort			Validation Cohort		
		Non-AHF Group (*n* = 244)	AHF Group (*n* = 76)	*P* value	Non-AHF Group (*n* = 111)	AHF Group (*n* = 26)	*P* value
Demographics							
Gender, male, n (%)		188 (77.05%)	43 (56.58%)	**<0**.**001**	87 (78.38%)	16 (61.54%)	0.074
Age, years, mean ± SD		59.59 ± 13.13	71.46 ± 12.97	**<0**.**001**	59.59 ± 14.17	72.88 ± 14.88	**<0**.**001**
Medical history							
Hypertension, n (%)		149 (61.07%)	52 (68.42%)	0.247	66 (59.46%)	17 (65.38%)	0.578
Diabetes, n (%)		68 (27.87%)	24 (31.58%)	0.533	23 (20.72%)	6 (23.08%)	0.791
AF, n (%)		18 (7.38%)	41 (53.95%)	**<0**.**001**	10 (9.01%)	10 (38.46%)	**<0**.**001**
COPD, n (%)		15 (6.15%)	10 (13.16%)	**0**.**047**	10 (9.01%)	6 (23.08%)	0.095
VHD, n (%)		70 (28.69%)	66 (86.84%)	**<0**.**001**	30 (27.03%)	21 (80.77%)	**<0**.**001**
BBB, n (%)		14 (5.74%)	9 (11.84%)	0.072	3 (2.70%)	7 (26.92%)	**<0**.**001**
Systolic function, n (%)		4 (1.64%)	13 (17.11%)	**<0**.**001**	2 (1.80%)	4 (15.38%)	**0**.**012**
Diastolic function, n (%)		62 (25.41%)	35 (46.05%)	**<0**.**001**	30 (27.03%)	14 (53.85%)	**0**.**008**
St segment, n (%)		58 (23.77%)	36 (47.37%)	**<0**.**001**	19 (17.12%)	12 (46.15%)	**0**.**001**
Clinical features							
Admission NIHSS, score, n (%)	Score subgroup			**<0** **.** **001**			**0**.**006**
	1	67 (27.46%)	10 (13.16%)		38 (34.23%)	2 (7.69%)	
	2	142 (58.20%)	44 (57.89%)		60 (54.05%)	16 (61.54%)	
	3	35 (14.34%)	22 (28.95%)		13 (11.71%)	8 (30.77%)	
Admission GCS, score, n (%)	Score subgroup			**0** **.** **003**			**0**.**045**
	1	15 (6.15%)	9 (11.84%)		10 (9.01%)	4 (15.38%)	
	2	37 (15.16%)	20 (26.32%)		11 (9.91%)	7 (26.92%)	
	3	192 (78.69%)	47 (61.84%)		90 (81.08%)	15 (57.69%)	
Admission mRS, score, n (%)	Score subgroup			**<0** **.** **001**			**0**.**004**
	0	135 (55.33%)	60 (78.95%)		65 (58.56%)	23 (88.46%)	
	1	109 (44.67%)	16 (21.05%)		46 (41.44%)	3 (11.54%)	
Inspection results							
Hemoglobin, g/L, mean ± SD		128.20 ± 20.41	117.42 ± 19.83	**<0**.**001**	129.17 ± 18.32	114.08 ± 20.07	**<0**.**001**
SBP, mmHg, mean ± SD		149.95 ± 22.75	148.57 ± 25.72	0.653	149.85 ± 20.13	151.73 ± 27.29	0.848
DBP, mmHg, mean ± SD		88.29 ± 13.30	85.42 ± 14.09	0.107	89.06 ± 12.34	90.27 ± 21.17	0.668
K, mmol/L, mean ± SD		4.01 ± 0.50	4.14 ± 0.57	0.057	3.98 ± 0.39	4.13 ± 0.71	0.510
HDL, mmol/L, mean ± SD		0.99 ± 0.25	1.07 ± 0.44	0.178	1.07 ± 0.30	1.09 ± 0.31	0.794
LDL, mmol/L, mean ± SD		2.68 ± 0.86	2.41 ± 0.89	**0**.**021**	2.72 ± 0.76	2.60 ± 1.06	0.365
EF,%, median (IQR)		67.50 (63.00–72.00)	62.00 (56.00–66.00)	**<0**.**001**	66.00 (62.00–72.00)	64 (52.50–68.50)	**0**.**034**
CRP, mg/L, median (IQR)		65.12 (2.13–14.86)	11.11 (2.37–30.62)	**0**.**031**	4.28 (2.32–9.66)	6.52 (1.97–25.85)	0.423
D-dimer, μg/mL, median (IQR)		0.52 (0.26–1.49)	1.05 (0.50–2.49)	**<0**.**001**	0.50 (0.27–1.15)	1.62 (0.69–4.00)	**<0**.**001**
Creatinine, μmol/L, median (IQR)		76.50 (63.15–91.00)	92.65 (67.48–134.02)	**<0**.**001**	74.90 (63.00–90.00)	104.00 (74.53–132.05)	**<0**.**001**
cTnT, ng/mL, median (IQR)		0.008 (0.006, 0.012)	0.009 (0.006, 0.014)	0.1565	0.009 (0.006, 0.012)	0.009 (0.006, 0.013)	0.8727

AF, atrial fibrillation; COPD, chronic obstructive pulmonary disease; VHD, valvular heart disease; BBB, bundle branch block; St, ST Segment[an important wave segment in an electrocardiogram (ECG)]; SBP, systolic blood pressure; DBP, diastolic blood pressure; HDL, high-density lipoprotein; LDL, low-density lipoprotein; EF, ejection fraction [a parameter of transthoracic echocardiography (TTE) for evaluating cardiac pumping function]; CRP, C-Reactive Protein; cTnT, cardiac troponin T.

*P* values in bold indicate *P* < 0.05.

### 3.3 Development and validation of the predictive nomogram

#### 3.3.1 Predictors selection and model construction

**Figure 2:**
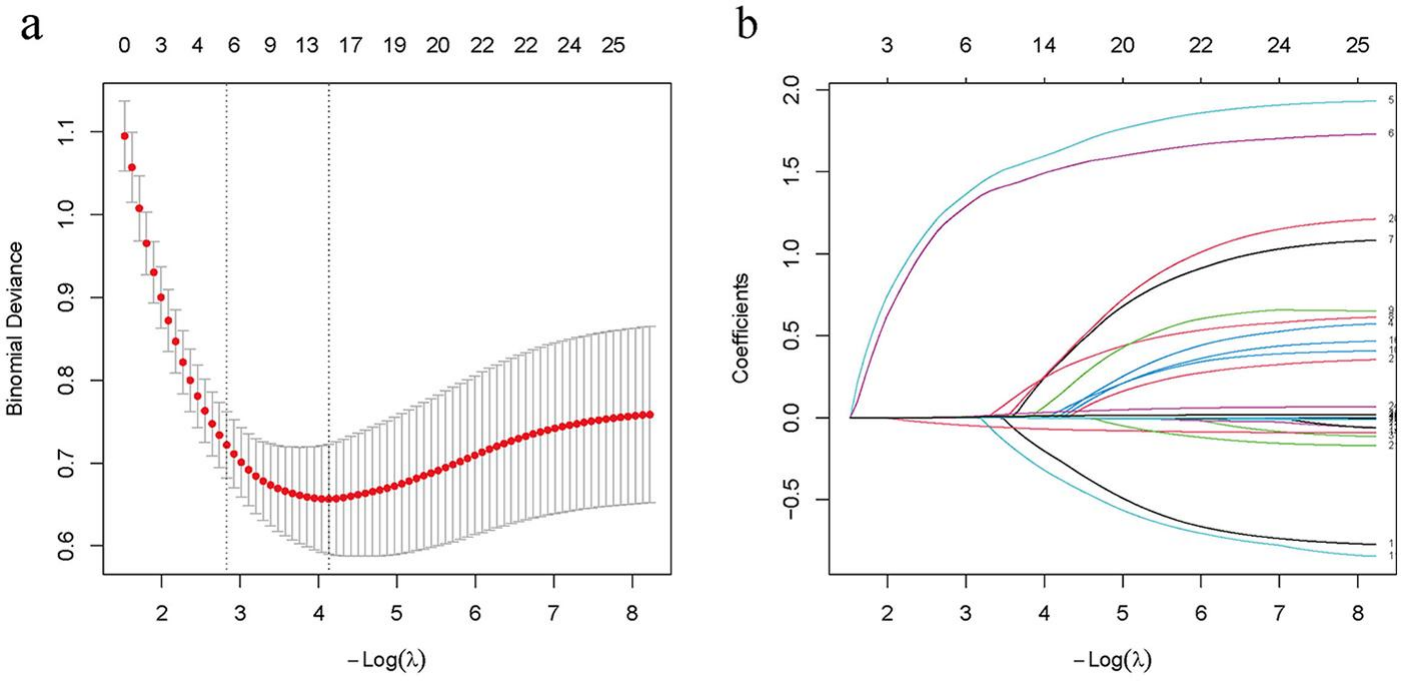
LASSO regression analysis for posT,stroke AHF feature selection. **(a)** Binomial deviance versus −log(*λ*). The displays two vertical dashed lines representing values determined through 10-fold cross-validation. The horizontal axis shows the negative logarithmic transformation of the optimal penalty parameter [–log(*λ*)], where *λ*. min (minimum deviation criterion) corresponds to –log(*λ*) = 4, whereas *λ*.1se (1-standard-error criterion) corresponds to –log(*λ*) = 3. The vertical axis depicts the binomial deviance obtained from cross-validation. **(b)** LASSO coefficient profiles. This illustrates the trajectories of the LASSO coefficients for all 24 variables across a sequence of log-transformed *λ* values [–log(*λ*)]. Based on the optimal *λ* = 0.05, five variables with nonzero coefficients were retained.

LASSO regression identified risk factors predictive of incident post-stroke AHF in the training cohort. The ideal regularization value was found to be *λ* = 0.05 (*λ*.1se criterion) using 10-fold cross-validation, with parameter selection optimized to achieve the minimum standard error. From the 24 candidate variables, five predictors most closely associated with post-stroke AHF were ultimately selected: atrial fibrillation, valvular heart disease, ejection fraction, creatinine, and cardiac troponin T. Figures 2a,b provide a graphic depiction of these findings.

**Figure 3:**
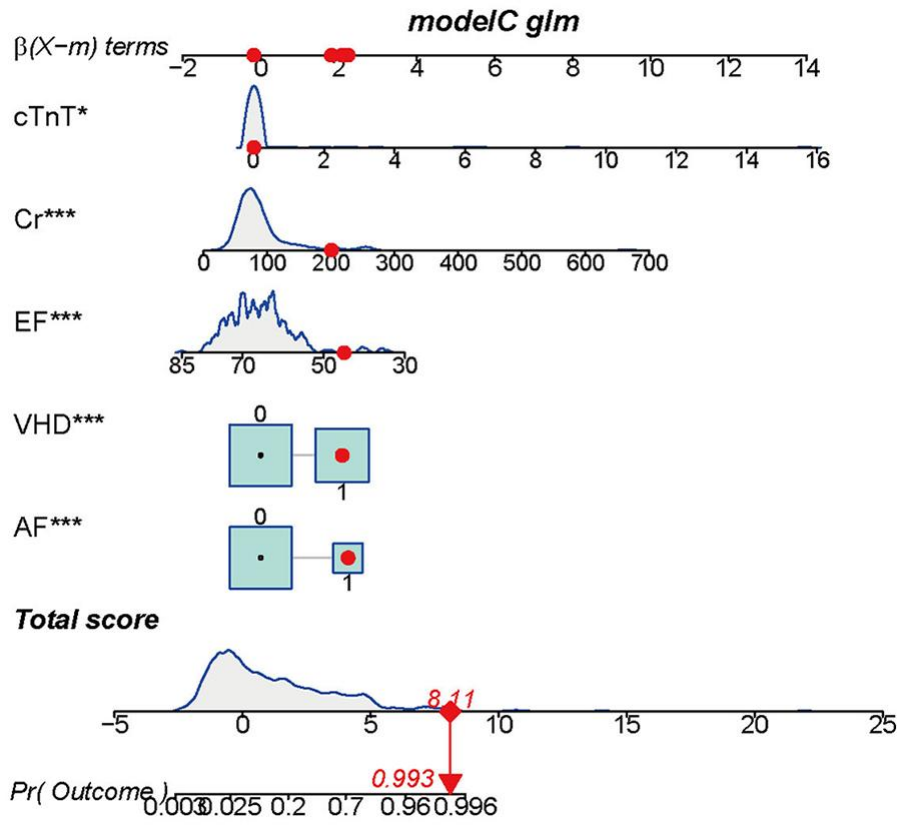
Nomogram of the posT,stroke EVT patients for AHF prediction. The dots represent the prediction results of patients. *** represents the highest level of significance (***p < 0.001) and * (*p < 0.05).

The final multivariate logistic regression analysis revealed that atrial fibrillation, valvular heart disease, ejection fraction, creatinine, and troponin T are independent predictors of acute heart failure after stroke surgery (Table 2). There were statistically significant differences for each of the five predicted variables. A risk nomogram for sudden heart failure following stroke surgery was created based on these five variables (Figure 3).

**Table 2:** Results of multivariable logistic regression of AHF after EVT.

Variables		OR(95%CI)	P
AF	No	Reference	<0.001
	Yes	8.569 (4.360,16.842)	
VHD	No	Reference	<0.001
	Yes	8.463 (4.313,16.606)	
EF		0.897 (0.860, 0.935)	<0.001
cTnT		2.469 (1.065, 5.721)	0.035
Cr		1.013 (1.006,1.020)	<0.001

AF, atrial fibrillation [a parameter of transthoracic echocardiography (TTE) for evaluating cardiac pumping function]; VHD, valvular heart disease; BBB, bundle branch block; EF, ejection fraction; cTnT, cardiac troponin T; Cr, serum creatinine.

#### 3.3.2 Performance and validation of the nomogram

**Figure 4:**
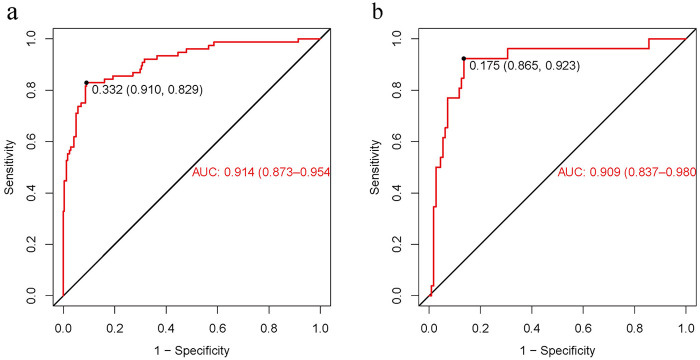
ROC curves for the AHF predictive nomogram in AIS, LVO patients after EVT. **(a)** In the training cohort. The area under the AUROC was 0.913 (95% CI: 0.873–0.953). At a risk probability cut-off of 0.331, the model showed a specificity of 0.910 and sensitivity of 0.829. **(b)** For the validation cohort. The AUROC was 0.909 (95% CI: 0.837–0.980). At a cutoff of 0.175, specificity reached 0.865 with a sensitivity of 0.923.

**Figure 5:**
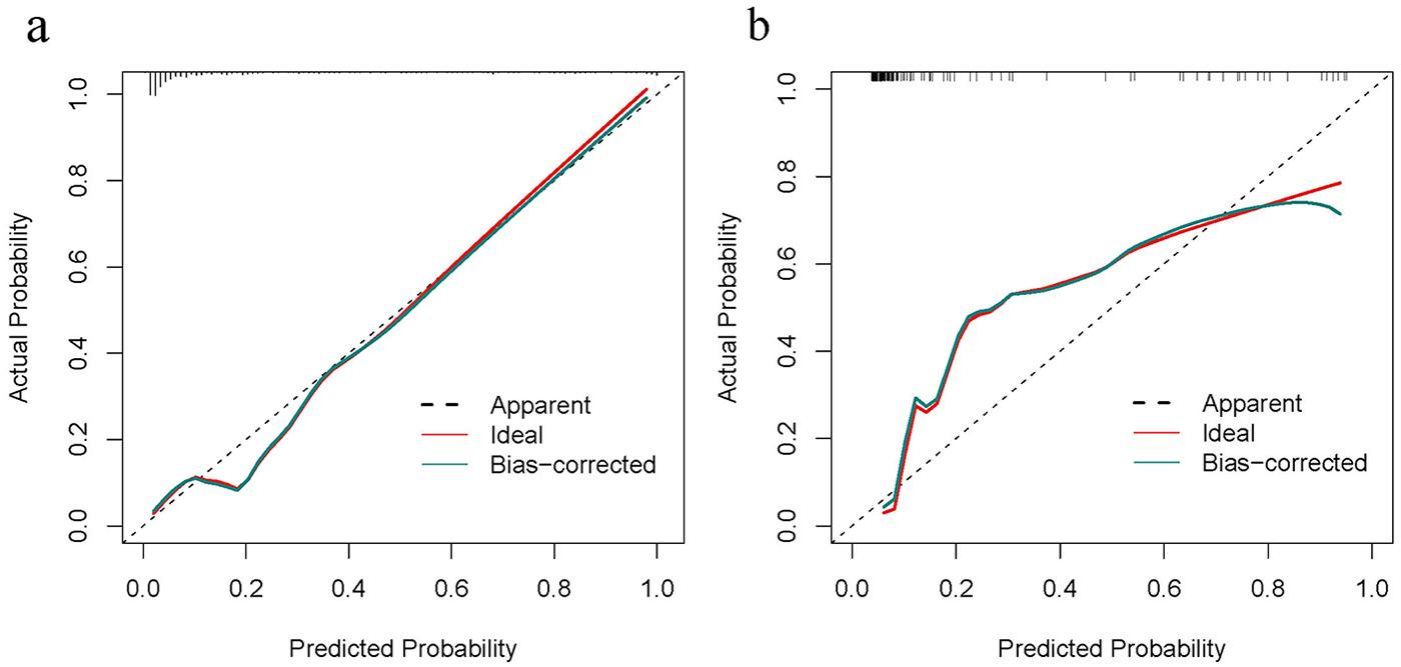
The calibration curves for the AHF nomogram in AIS,LVO patients after EVT. **(a)** The training cohort. **(b)** The validation cohort. The diagonal dashed line denotes the benchmark prediction of a perfectly calibrated model, and the solid line represents the probability forecasts derived from the nomogram. The level of congruence between predicted and real outcomes is evaluated by the closeness of the solid to the dashed line; minimal separation indicates superior calibration performance. The calibration intercept is 0 and the calibration slope is 1 for both the training set and the validation set.

The model's predictive ability was evaluated using the ROC curve. In the training cohort, the area under the ROC curve (AUROC) was 0.913, while in the validation cohort, the AUROC was 0.909 (Figures 4a,b), indicating that the nomogram model demonstrated robust discriminative capacity. Calibration accuracy and the calibration curves were quantified via the Hosmer-Lemeshow GOF test (Figures 5a,b), the HL test was performed using 10 groups with 8 degrees of freedom. The results showed *P* = 0.189 in the training cohort and *P* = 0.414 in the validation cohort, demonstrating close calibration of predicted to observed AHF rates across both cohorts. Internal validation was performed using 500 bootstrap resamples in the training cohort to assess model discrimination and calibration, further demonstrating good overall predictive accuracy and calibration performance (Supplementary Figures S2, S3).

**Figure 6:**
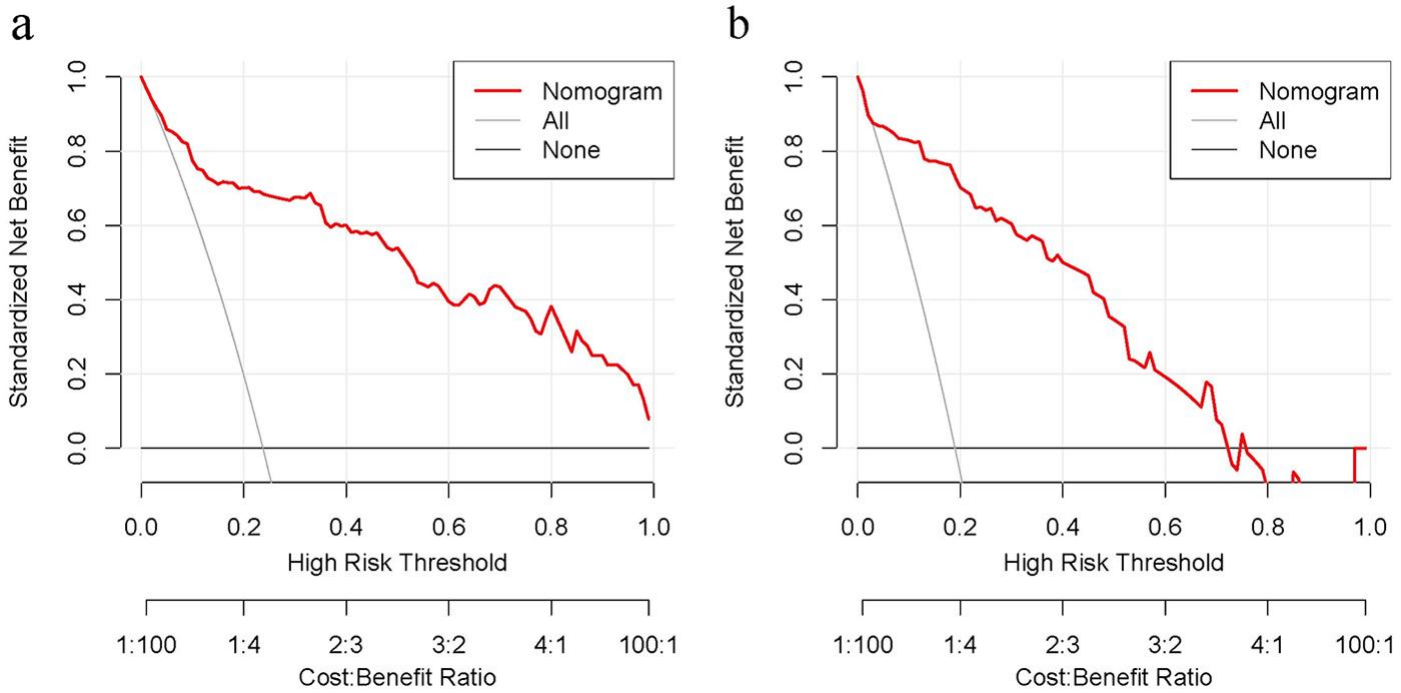
The DCA curves illustrate the performance of the AHF prognostic nomogram in AIS, LVO patients following EVT. The red curve (Nomogram) represents the nomogram model constructed in this study, reflecting its standardized net clinical benefit at different high-risk thresholds. The black horizontal line (“No intervention” strategy) serves as a benchmark reference for clinical decision-making, while the gray diagonal line (“Full intervention” strategy) represents an extreme decision-making mode without risk stratification. **(a)** The training cohort. **(b)** The validation cohort. A risk threshold range of 5%–80% in the training cohort and 5%–70% in the validation cohort, the model demonstrates superior clinical net benefit compared with both reference curves.

**Figure 7:**
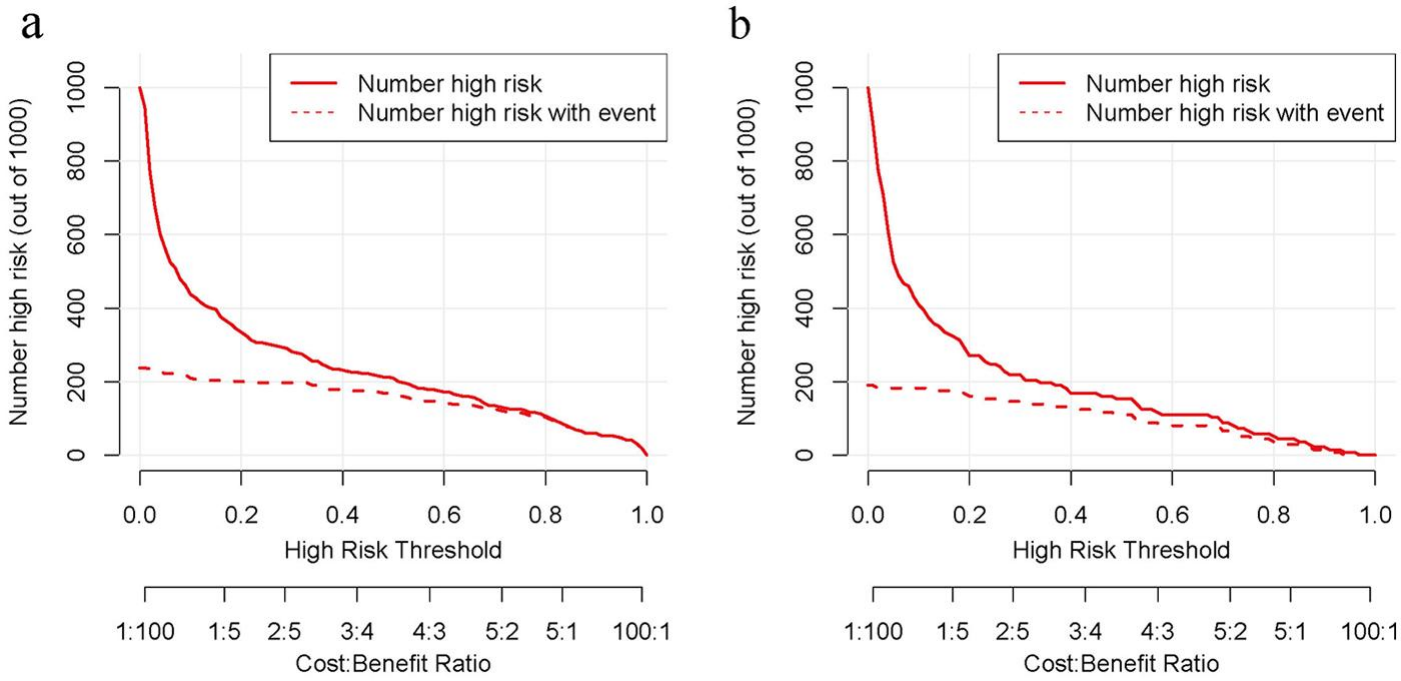
The CIC analysis for the AHF predictive nomogram in AIS-LVO patients is illustrated. **(a)** The training cohort. **(b)** The validation cohort. The number of patients at high risk of AHF per 1000 patients is shown on the vertical axis. The horizontal axis represents the probability threshold for determining patients as being at high risk of AHF. “Cost: Benefit Ratio” refers to the cost-benefit ratio. High-risk AHF patients (solid red line) versus those with actual AHF events (dashed red line).

The clinical usefulness of the nomogram was evaluated using DCA (Figures 6a,b). When the predicted risk of AHF exceeds approximately 5%–40%, clinicians may consider initiating additional cardiac monitoring measures. Furthermore, CIC were produced in order to evaluate the model's clinical influence (Figures 7a,b). The results indicated that when the risk threshold exceeded 0.80, the actual incidence of the disease roughly matched the number of anticipated positive events.

## 4 Disscusion

The accelerated aging of the population and urbanization have led to the increasing prevalence of unhealthy lifestyles, subsequently widening the exposure to cardiovascular and cerebrovascular disease risk factors. Stroke, as an acute and severe manifestation of cardiovascular and cerebrovascular diseases, is witnessing a concerning rise in incidence (18). Although EVT has become a core method for revascularization in patients with acute large vessel occlusion ischemic stroke, significantly reducing mortality and disability rates, postoperative AHF remains a critical complication influencing patient prognosis. As an acute and severe condition in neurological intensive care, AHF is closely associated with prolonged hospitalization, increased consumption of medical resources, and a higher mortality rate within three months post-stroke (6). This study, through systematic clinical data collection and rigorous statistical analysis, is the first to construct and validate a predictive model for AHF after EVT in patients with AIS-LVO. This model fills a gap in risk prediction tools in this field and provides clinicians with a scientifically grounded and practical decision-making tool for preoperative assessment and early postoperative intervention.

Currently, the primary method used to diagnose AHF is symptoms, signs, laboratory tests, and imaging studies. However, these methods face significant limitations in EVT for AIS-LVO patients. Most of these patients undergo intubation or sedation post-procedure, making it difficult to accurately describe symptoms. Additionally, key laboratory markers such as NT-proBNP may be specifically reduced due to cerebral ischemic stress (19). A more prominent issue is that without early diagnosis of AHF, existing treatment strategies face serious limitations in critically ill neurological patients. Diuretics and positive inotropic agents, while aiming to alleviate heart failure symptoms, may adversely affect stroke recovery by reducing cerebral blood flow (20). A clinically deployable nomogram predicting AHF risk after EVT was developed and validated herein. Our results show that atrial fibrillation, valvular heart disease, left ventricular ejection fraction (LVEF), creatinine, and cardiac troponin T are strongly associated with post-stroke AHF. Leveraging these variables, we developed a clinically applicable predictive nomogram.

The model's strength lies in its focus on the unique pathophysiological mechanisms after EVT: brain-heart interactions (21), reperfusion injury-induced systemic inflammation (22), and the compounded stress on the heart and kidneys caused by cerebrovascular intervention (23, 24). Compared to the EVT complication prediction model (THRIVE score) proposed by Liu P et al. (25), our model is the first to focus on acute heart failure, a highly fatal complication, and utilizes LASSO regression for precise dimensionality reduction of predictive factors, thus avoiding biases from multicollinearity that are common in traditional regression models.

Multivariate logistic regression analysis revealed that atrial fibrillation is a key risk factor for post-stroke acute heart failure, with a higher risk weight compared to other causes. This finding is consistent with conclusions from several large-scale clinical studies in recent years (26), further solidifying atrial fibrillation's central role in the risk chain of stroke occurrence, recurrence, and postoperative complications. Atrial fibrillation not only directly causes cardiogenic embolic ischemic stroke but also induces sustained hemodynamic instability and systemic endothelial dysfunction (27, 28), which indirectly accelerates vascular lesion progression and dysfunction. These changes are more pronounced post-stroke, leading to the development of acute heart failure. As a result, an “atrial fibrillation - ischemic stroke - acute heart failure” vicious cycle is easily formed (21), significantly increasing the patient's risk of mortality and disability. Furthermore, atrial fibrillation, heart failure, and stroke share similar risk factors (29), emphasizing the importance of managing atrial fibrillation in post-stroke patients (30). It is recommended that patients with atrial fibrillation avoid alcohol and maintain a healthy weight (31), as well as control ventricular rhythm and avoid medications that could interfere with atrial fibrillation management.

Valvular heart disease (VHD) in this nomogram is positively correlated with AHF risk and is also a risk factor for atrial fibrillation. Our multivariate regression analysis showed that valvular heart disease increases AHF risk by threefold. VHD is one of the most common underlying diseases associated with AHF (32), as acute hemodynamic stress from chronic valvular disease or significant new valvular lesions can precipitate AHF (33). Hemodynamic fluctuations during stroke intervention can worsen systolic or diastolic dysfunction in patients with valvular disease (34), possibly related to persistent neurogenic myocardial injury and myocardial fibrosis (35). Particularly in patients with mitral stenosis, the sudden increase in pulmonary venous return after vascular reperfusion can lead to acute pulmonary edema. However, it is important to note that the current model does not distinguish between types of valvular diseases, and future research will refine the independent effects of aortic and mitral valve diseases.

The most popular metric for evaluating left ventricular systolic function is left ventricular ejection fraction (LVEF). As a key parameter of heart function, LVEF is significantly negatively correlated with the risk of AHF in the nomogram, indicating that a higher risk of AHF is linked to a lower LVEF. Studies have shown that patients with lower LVEF experience more frequent cardiovascular events within 180 days (36). The left ventricle is the primary chamber of the heart, and we hypothesize that impaired left ventricular function is related to heart failure risk. Our data also highlights the critical role of diastolic dysfunction in the occurrence of AHF: the proportion of AHF patients with diastolic dysfunction (46.05%) is significantly higher than that in non-AHF patients (25.41%). Diastolic dysfunction is often associated with increased myocardial stiffness and impaired active relaxation (37), involving inflammation-mediated myocardial fibrosis and calcium regulation abnormalities (38).

Cardiac troponin T (cTnT) in this model is positively correlated with the risk of AHF. cTnT is considered a highly specific and sensitive biomarker for detecting cardiac injury and left ventricular (LV) dysfunction (39). A recent study reported that high-sensitivity cTnT levels in acute ischemic stroke patients were significantly elevated, potentially triggering autonomic imbalance and sympathetic nervous system upregulation, leading to myocardial injury from necrosis or ischemia in the contraction zone (40). Wu Y et al. found that about 45.4% of large vessel occlusion stroke patients had elevated hs-cTnT levels (>14 ng/L) on admission (41), and multivariate logistic regression analysis showed that congestive heart failure was independently associated with elevated hs-cTnT levels. This phenomenon supports the “cardio-cerebral comorbidity” theory, in which neurological emergencies may induce secondary cardiac injury.

Elevated creatinine levels are positively correlated with the risk of AHF. Serum creatinine is widely used as a marker for evaluating renal function, and elevated levels reflect renal dysfunction, which is also associated with poor outcomes post-stroke (42). Our findings align with previous studies, showing that poorer renal function correlates with worse prognosis. Renal insufficiency leads to fluid retention and activation of the renin-angiotensin-aldosterone system (RAAS) (43), directly increasing cardiac load. It also results in the accumulation of uremic toxins, which have direct myocardial suppressive, pro-inflammatory, and pro-fibrotic effects, leading to systolic dysfunction and arrhythmias (44).

## 5 Conclusion

This study demonstrated significant associations of atrial fibrillation, valvular heart disease, reduced ejection fraction, elevated creatinine, and troponin T levels with acute heart failure in the post-stroke setting. Clinically, these factors are generally obtainable at the point of care. The nomogram may serve as a useful tool for estimating the risk of AHF after EVT, potentially helping clinicians identify patients at higher risk earlier and consider more tailored interventions. Given its relative simplicity and accessibility, the model appears to have translational potential and could, pending further external validation, complement existing decision-support systems in neurocritical care.

## 6 Limitations and future directions

First, this study adopted a single-center retrospective design, with data sourced from past cases at a single medical center, which inevitably introduces the risk of selection bias. There may also be missing key clinical data in the retrospective design. Furthermore, the internal validation of the model cannot fully avoid the risk of “overfitting” and may not reflect the model's adaptability in real clinical settings. As AHF adjudication relied in part on retrospective medical record review and previously documented clinical diagnoses, some degree of information bias may be unavoidable. In addition, the current sample size may have led to an overestimation of the model's discriminative performance, therefore, the AUC value should be interpreted as an exploratory estimate rather than evidence supporting direct clinical implementation. To further evaluate the stability and applicability of the model, external validation is required, with calibration and optimization of model parameters if necessary. Finally, while left ventricular ejection fraction (LVEF) is a core indicator for classifying heart failure, our study did not include LVEF classification, which may affect the precise risk stratification and intervention guidance for high-risk patients in clinical practice. Future studies could promote a multi-center design to optimize sample and data quality. The model could be enhanced by strictly following international guidelines to distinguish heart failure subtypes and incorporating LVEF classification as a stratification variable, exploring the mechanistic differences of these subtypes post-stroke.

## Data Availability

The clinical data in this study are available from the authors upon request. Requests to access these datasets should be directed to Xiuling Yang, 3260677031@qq.com.
