# Supplementary material for: Risk assessment of acute heart failure after endovascular therapy in acute ischemic stroke: a nomogram-based study
Source: Front Cardiovasc Med. 2026 Jul 20;13:1836280. doi: 10.3389/fcvm.2026.1836280 (PMC13429435; doi:10.3389/fcvm.2026.1836280)

**Figure S1**: Variable Missingness.


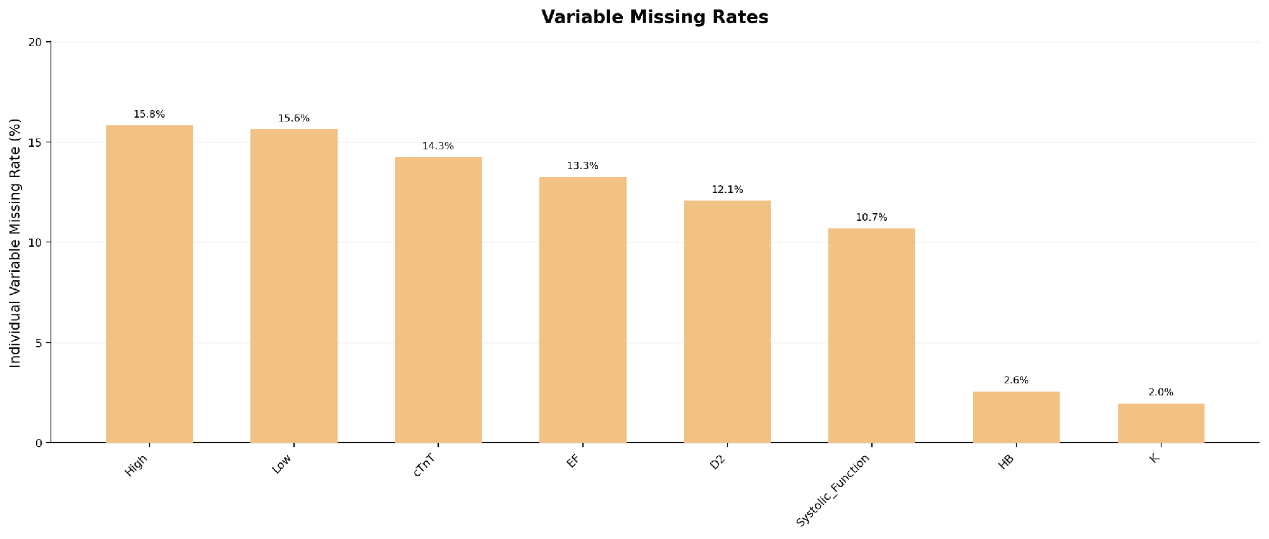


**Abbreviations**: **High**, High-Density Lipoprotein; **Low**, Low-Density Lipoprotein.

**Figure S2**: 500 bootstrap internal validation for ROC curves.


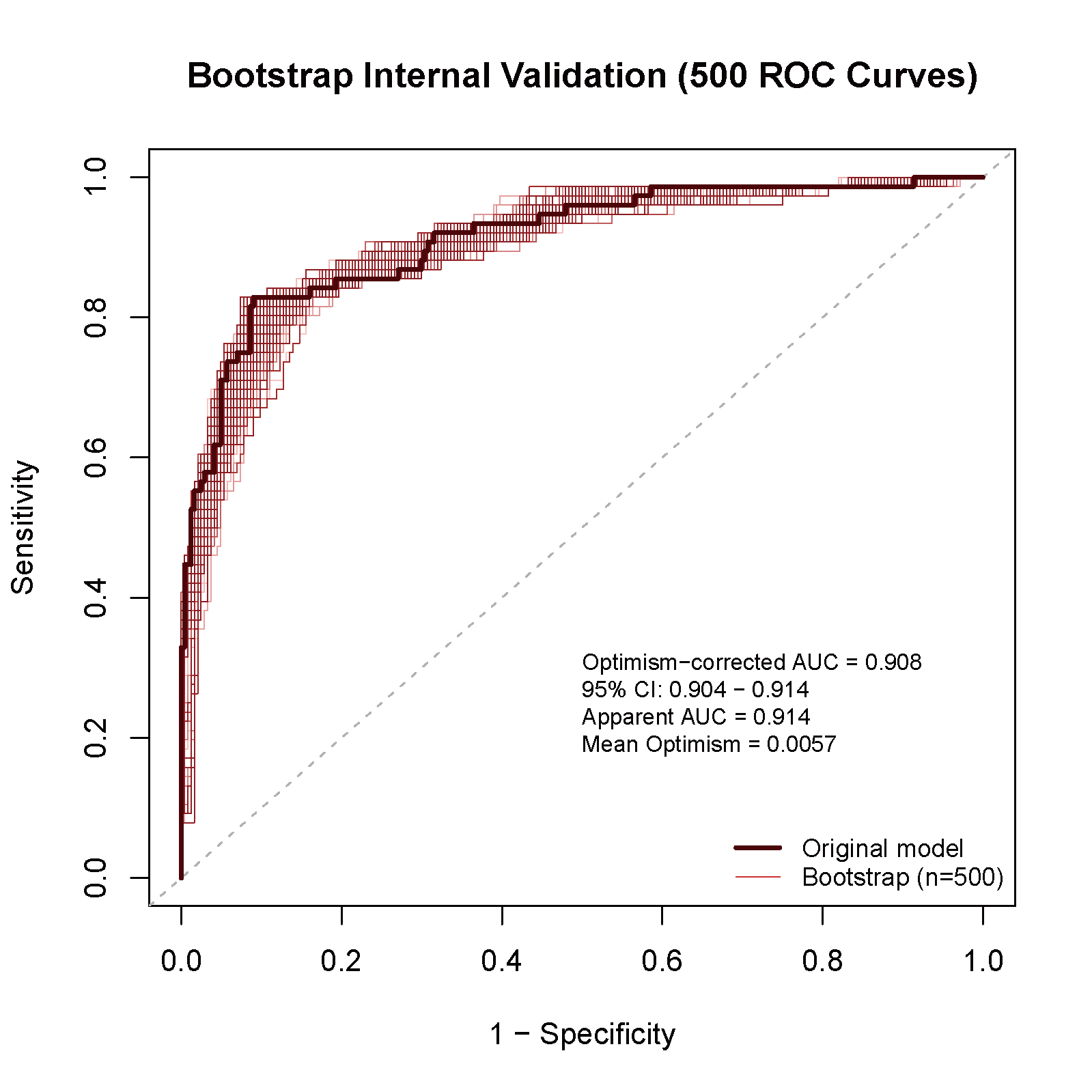


**Figure S3**: 500 bootstrap internal validation for calibration curves.


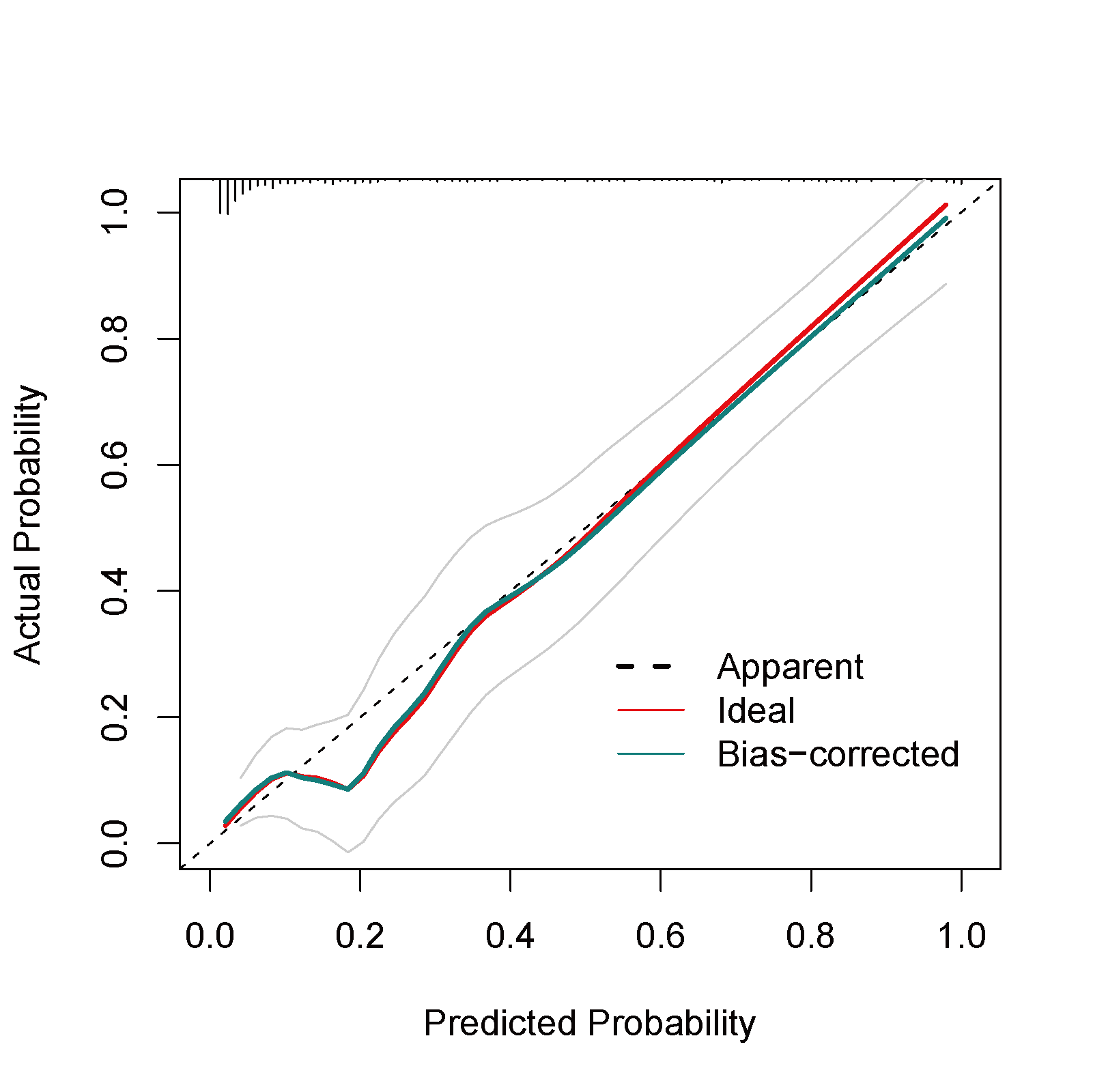

Supplement: Supplementary file 1 [file Datasheet1.docx]
